# Therapy of pyogenic liver abscess with a primarily unknown cause

**DOI:** 10.1007/s00423-022-02535-3

**Published:** 2022-05-30

**Authors:** Phil Meister, Hannes Irmer, Andreas Paul, Dieter P. Hoyer

**Affiliations:** Department of General-, Visceral-, and Transplantation Surgery, University Hospital Essen, Essen, Germany

**Keywords:** Liver abscess, Hepatology, Drainage hepatectomy

## Abstract

**Purpose:**

Pyogenic liver abscess (PLA) is a collection of pus in the liver, often without a known direct cause. There is discord on the best diagnostic and therapeutic strategy. We aimed to examine these questions in our patient cohort.

**Methods:**

A total of 66 out of 309 patients with PLA at our tertiary referral center between 2012 and 2020 had a primarily unknown cause. We analyzed PLA configuration, comorbidities, and whether an underlying cause could be found later. Therapy was sorted by antibiotics alone, percutaneous drainage, and primary surgery. Success was assessed by a change of initial therapy, in-hospital mortality, and mean hospital stay.

**Results:**

Overall mortality was 18%; in 55%, a causative condition could be found. CRP, GGT, size, and multiple localization go along with higher mortality. Antibiotics alone had a failure rate of 82%. Percutaneous drainage was successful in 70% of cases. Surgery was mainly reserved for failed previous non-surgical treatment and had in-hospital mortality of 12%.

**Conclusions:**

PLA goes along with high mortality. In the majority of all patients, a causative condition can be identified by detailed diagnostics. Percutaneous drainage together with antibiotics is the therapy of choice and is successful in 70% of cases. If drainage is insufficient or impossible, surgery is an effective alternative.

## Introduction

A hepatic abscess is defined as an intraparenchymal collection of pus. In case of microbiological infection, the diagnosis of pyogenic liver abscess (PLA) can be made. There are regional differences in incidence ranging from around 2/100,000 hospital admissions in the western world up to 270/100,000 in Taiwan [1]. Mortality is high and is described above 10% in literature [2].

A PLA can originate from a variety of causes. While appendicitis was a common cause in earlier days, bile stones and other biliary diseases are the utmost reason nowadays [3]. Medical and surgical procedures can often generate PLA as short- or long-term-complications, such as upper gastrointestinal or liver surgery [4], endoscopic retrograde cholangiography [5], radiofrequency ablation [6], or transarterial chemoembolization [7]. Also, intrahepatic neoplasia can often present as PLA and is only secondarily diagnosed as, e.g., hepatocellular carcinoma [1]. The cause of many other PLA remains unclear. There are indications a damaged intestinal barrier might promote hematogenic dissemination through the mesenteric-portal veins to the liver. Possible reasons for this damage are previous diverticulitis [8], colon cancer [9], or chronic inflammatory bowel disease [10]. Other risk factors are liver cirrhosis [11], diabetes mellitus [12, 13], and proton pump-inhibitor use [14].

There is no standard classification of PLA. Some authors suggest a differentiation in infectious, malignant, and iatrogenic genesis [15]. Especially the graduation in malignant PLA is vital due to possibly different therapeutic approaches [2]. In our eyes, the suggested graduation does not stand helpful in clinical practice but rather in scientific discord. As a tertiary referral center, we tend to see patients who develop a PLA with no recognizable cause. We want to distinguish between PLA with a recognizable medical condition or previous intervention leading to the PLA and primarily unknown PLA without an initially determinable cause as diagnostic management may vary.

There are several treatment options for PLA. The keystone is antibiotic treatment which all patients should receive. Another therapeutic option is drainage of the PLA to evacuate the trapped pus. Drainage can be performed radiologically guided via sonography or computer tomography. Insertion of a drain and singular aspiration are both viable options. Alternatively, drainage can be received through surgery. Surgical treatment is performed in ruptured abscess or failed conservative therapy. Resection of the affected segment or lobe can be a promising therapy in these cases [16].

Hope et al. developed a treatment protocol in 2008 based on a retrospective analysis of 107 patients over 7 years to determine the optimal treatment for PLA [17]. Singular small PLA below 3 cm diameter should receive antibiotics alone, while larger PLA should be drained interventionally. Complex PLAs with a multilocular presentation were suggested for primary surgical treatment.

Recommendations for PLA treatment are controversial, and the latter proposed protocol contradicts the more recent literature, especially in terms of surgical treatment.

Therefore, we aim to analyze our patient cohort with primarily unknown PLA to specify optimal therapeutic management for PLA further.

## Materials and methods

Data of all patients with the diagnosis of a liver abscess treated in the University Hospital of Essen between 01.01.2012 and 31.12.2020 were extracted from the digital hospital information system. Two independent investigators verified every patient’s diagnosis in the next step, and only patients with primarily unknown PLA were further included in this study. Sixty-six of 309 patients (21.4%) were suffering from a primarily unknown PLA; most others had a hepatic abscess immediately after surgery or other medical interventions. Those patients were not further considered for this study. This retrospective study was approved by the local ethics committee (21–10,227-BO) and followed the Declaration of Helsinki.

Patient characteristics such as gender, age, and comorbidities at the date of diagnosis were noted. Furthermore, the abscess properties like size, localization, and formation (like multiple lesions), presence of conditions promoting liver abscess (previous diverticulitis, malignoma, or immune suppression), and microbiological colonization were included. Etiology was by definition initially unclear but possibly diagnosed later and then grouped into 4 groups: “cholestatic” as a result of bile duct obstruction, “vascular” as result of a thromboembolic or obstructive event in the liver vessels, “malignoma” when directly associated with a liver tumor, and “infectious” after hematogenic dissemination from an infectious focus like diverticulitis. In some cases, the abscess’s origin remained cryptic. Lab values like leukocyte count, C-reactive protein (CRP), liver aminotransferases, international normalized ratio (INR), and creatinine were analyzed (Table 1).

**Table 1:** Patient characteristics of patients with a primary liver abscess at the date of diagnosis

***N* = 66**	
**Age**, mean (min–max)	55.91 (13–89) years
**Sex**, male	36 (54.5%)
**Comorbidities**	
Diabetes	11 (16.7%)
Cirrhosis	3 (4.5%)
Malignoma	10 (15.2%)
Immunosuppression	3 (4.5%)
Previous diverticulitis	6 (9.1%)
Previous pancreatitis	8 (12.1%)
**Abscess properties**	
Size, mean (min–max)	6.5 (1.5–18) cm
Multiple lesions	23 (34.8%)
**Abscess etiology**	
Infectious	20 (30.3%)
Vascular	4 (6.1%)
Cholestatic	7 (10.6%)
Malignoma	6 (9.1%)
Unknown	29 (44.6%)

The clinical treatment decision was then used to group the patients: conventional treatment with antibiotics only, additional interventional treatment with percutaneous drainage, or additional surgical treatment with primary surgery. The initial intention had to be clearly documented to be considered for analysis. The success was measured by the length of hospitalization and in-hospital mortality, while treatment failure was determined as a change of chosen, initial treatment regimen, or occurrence of major complications (Dindo-Clavien > 3).

Retrospectively adherence to the proposed treatment strategy [17] was checked, and the patients’ outcome was analyzed.

### Statistics

Data were analyzed using SPSS 27.0 software (IBM Inc., Armonk NY, USA). A two-sided *t* test was performed in comparison of mean values and the 95% confidence interval (CI) given. Data are given as mean values with standard deviation or with median and range as appropriate. ANOVA was performed in multigroup comparison. Binary logistic regression analysis was used to determine risk factors and other dependencies. A *p* value of < 0.05 was considered statistically significant.

## Results

Patients of all ages and equally both genders developed PLA. Only diabetes and malignoma as known risk factors were observed in barely 15% of patients, while immunosuppression and cirrhosis were rarely present. Abscesses had a mean size of 6.5 cm, whereas 89.4% were larger than 3 cm. Etiology remained unclear in 44.6% of patients. The most common cause in the remaining patients was infectious dissemination (30.3%) after, e.g., pancreatitis (12.1% of all patients) or diverticulitis (9.1% of all patients). Vascular, cholestatic, or malignant causes were equally distributed on the remaining 25% of patients.

### Laboratory values (Table 2)

**Table 2:** Lab values at admission in our center of patients with primary liver abscess. Mean values are displayed in the middle column, while the right column shows the percentage of patients with pathological findings for each lab value. The norm is noted right next to it. *WBC*, white blood cell count; *AST*, aspartate-aminotransferase; *ALT*, alanine-aminotransferase; *GGT*, gamma-glutamyltransferase; *INR*, international normalized ratio; *CRP*, C-reactive protein

Parameter (unit)	Mean (min–max)	Pathological [norm]
WBC (× 10^9/l)	15.63 (2.2–53)	54.5% [> 10 × 10^9/l]
Bilirubin (mg/dl)	1.04 (0.2–8)	10.6% [> 2 mg/dl]
AST (U/l)	89.5 (11–799)	40.9% [> 50 U/l]
ALT (U/l)	77.1 (12–597)	34.8% [> 50 U/l]
GGT (U/l)	237 (18–1342)	80.3% [> 50 U/l]
INR	1.19 (0.9–2.1)	31.8% [> 1.2]
CRP (mg/dl)	16.1 (< 0.5–51)	97% [> 0.5 mg/dl]
Creatinine (mg/dl)	1.11 (0.3–3.7)	10.6% [> 1.8 mg/dl]

Almost all patients presented elevated C-reactive protein as a marker of systemic inflammation (97%), in contrast only half of the patients had a pathological white blood cell count. Pathological findings in gamma-glutamyltransferase (GGT) (80.3%) were rather common, while only a third of patients showed pathological liver aminotransferases or INR values.

### Microbiological findings

Results from both drainage material and blood cultures were considered for this study. Many patients had no positive microbiological findings (52%) (often due to early antibiotic therapy). 34.8% had multiple microbiological pathogens. In 27% of patients, gram-negative germs and, in 21% of patients, gram-positive germs were detected, while 7.5% showed both types of pathogens. A mycotic infection could be found in 6% of patients, always together with gram-negative pathogens. There was no significant aggregation of a particular pathogen in our cohort.

### Therapy

Twenty-eight patients received primary conservative treatment (42.4%), and 34 patients received primary interventional treatment (51.5%). Four patients had surgery as primary treatment of liver abscess (6.1%). The therapy of 51.5% of patients was escalated in their clinical course. Reasons for escalation were clinical and laboratory non-response. An overall of 23 (34.8%) patients had major complications (Dindo-Clavien > 3), while a therapy escalation was not counted as a complication. The mean hospital length of stay was 40.6 days. Twelve (18.2%) patients died in hospital during their abscess therapy. For detailed therapy results sorted by regimen, see Table 3.

**Table 3:** Abscess therapy and its success sorted by initial therapy regimen. Therapy escalation was marked as a clinical decision for a therapy step up to a more invasive procedure. Major complications do not include a therapy escalation but unexpected major complications (Dindo-Clavien > 3)

*n* = 66	Conservative	Interventional	Surgery
*n* = (%)	28 (42.4%)	34 (51.5%)	4 (6.1%)
Adherence to therapy scheme [17]	3 (10.7%)	24 (70.6%)	0 (0%)
Therapy escalation	23 (82.1%)	11 (32.4%)	-
Escalation, if adherent	2 (67%)	6 (25%)	-
Mean hospital stay	62 ± 142.5	25 ± 28.78	18 ± 7.9
Major complications	7 (25%)	15 (44.1%)	1 (25%)
In-hospital mortality	5 (17.9%)	7 (20.6%)	0

### The suggested therapy protocol

Retrospectively assessed adherence to the proposed therapy protocol by Hope et al. was 48.5%. If adherent to the protocol, fewer patients (21.9%, 0 = 0.001) had to be stepped up in the course of their therapy, but had no different mortality, number of complications, or mean hospital stay.

### Conservative versus primary drainage

Patients who received conservative treatment first had to be stepped up in 82.1% of cases. Of these patients, 65% received secondary drainage, and 35% secondary surgery. In the interventional group, only 32.4% had to be upgraded (*p* = 0.001). Mean hospital stay had a trend to be longer (*p* = 0.17) in the conservative group, but there was no difference in in-hospital mortality (*p* = 0.79). There were no differences in size or lab values between these two therapy groups, but multiple abscess localization was found more often in the conservative treatment group (50% vs. 26%, *p* = 0.06). Patients with successful conservative treatment tended toward smaller abscess (2.5 cm vs. 3.1 cm mean diameter, *p* = 0.08) and lower liver aminotransferases (24 U/l vs. 143 U/l, *p* = 0.04) compared to failed conservative treatment. There was no difference in terms of multiple lesions (*p* = 0.64). In the primary drainage group, larger sized (5.8 cm vs. 7.8 cm mean diameter, *p* = 0.04) and multiple lesion PLAs (17% vs. 45% *p* = 0.08) showed a trend to therapy failure. The drainage group appears to present major complications more frequently than conservative therapy (44% vs. 25%, *p* = 0.10).

### Patients receiving surgery

An overall of 25 patients received surgery for their PLA (38%). Of note, only four were clinically selected for primary surgery, and 21 patients were operated due to failure of their previous therapy. Thirteen patients (52%) received surgical abscess drainage, and 12 patients (48%) had liver resection of the affected segment or lobe. Three patients died, all after surgical drainage. Patients selected for surgery had a larger abscess (7.4 cm vs. 5.9 cm mean diameter, *p* = 0.04) but no other significant differences in their characteristics. Mortality after primary or secondary surgical therapy was 12%, but 22% for the group of non-surgically treated patients (*p* = 0.29, not significant).

### Small singular abscess and multiple lesions

Only four patients had a singular PLA below 3 cm diameter, of which 3 received primary conservative treatment. Such an approach was successful in one patient. The other two had to receive drainage later. One patient was treated by primary drainage, which had to be escalated to surgery without avoiding patient lethality.

All patients with multiple lesion PLA (*n* = 23, 34.8%) received primary non-surgical treatment with 9 patients requiring surgery later (39%). Three patients died during conservative treatment (21%), and two more patients died after receiving surgery (22%). The success rate for non-surgical treatment in multiple PLA was 48%, and overall mortality in patients with multiple lesions was 22%.

### Regression analysis for survival (Fig. 1)

**Fig. 1:**
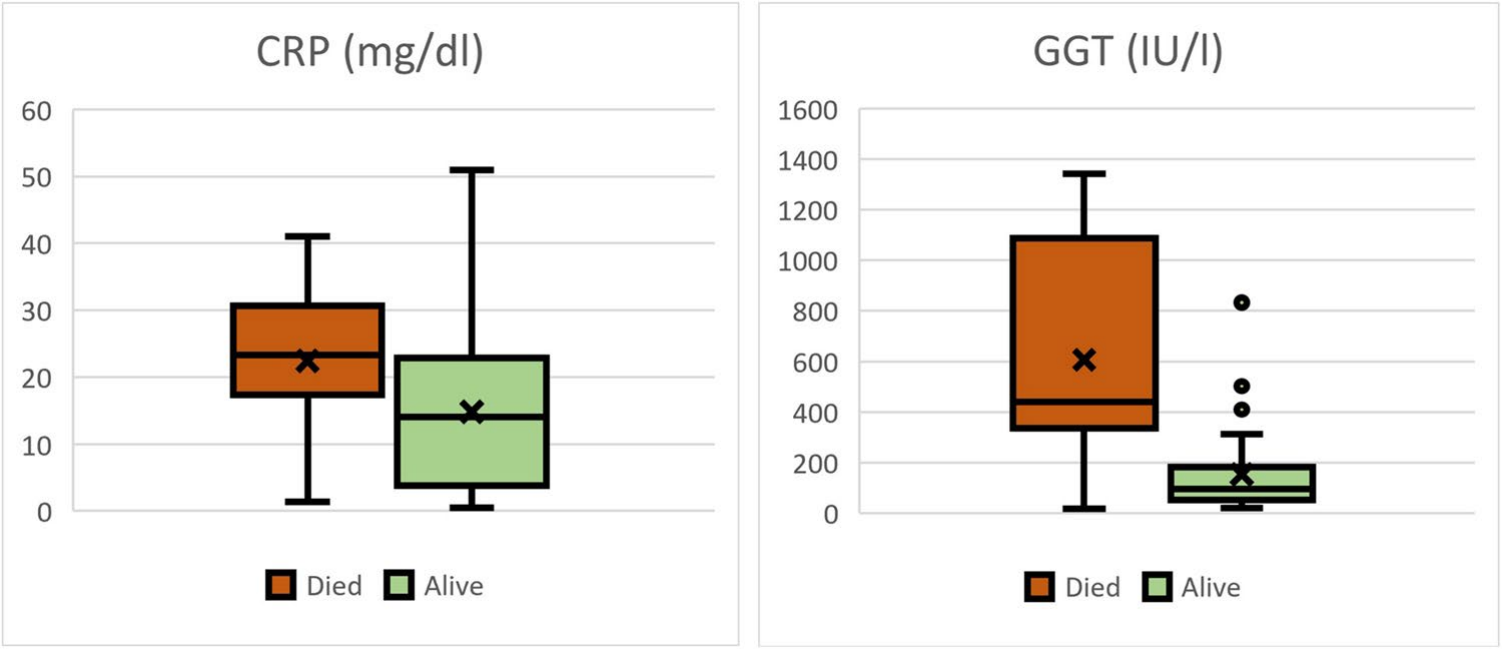
Boxplots for C-reactive protein and gamma-glutamyltransferase in dependence of PLA mortality. Patients who died from PLA had higher CRP (*p* = 0.03) and GGT (*p* = 0.004) than those who survived

Patients who died from their PLA had significantly higher CRP (22.5 mg/dl vs. 14.6 mg/dl, *p* = 0.03) and GGT (608 U/l vs. 154 U/l, *p* = 0.004); the differences in the other characteristics were not statistically significant.

A univariate binary logistic regression analysis was performed to identify risk factors for mortality in patients with liver abscesses. Multiple germs (OR 5.0, *p* = 0.018), mycotic infection (OR 17.7, *p* = 0.018), hyperbilirubinemia (OR 1.84, *p* = 0.018), and elevation of liver aminotransferases (e.g., AST > 50U/L, OR 6.0, *p* = 0.014) were identified as risk factors for mortality. For the detailed regression analysis, see Table 4.

**Table 4:** Univariate regression analysis for in-hospital mortality when suffering from a primary liver abscess. Significant findings were marked by bold type

Variable	OR	KI	*p*
Age	1.04	1.0–1.1	0.073
Male sex	0.80	0.23–2.80	0.727
Size	1.02	0.83–1.25	0.872
Multiple	1.43	0.40–5.13	0.585
Multiple germs	**5.00**	**1.31**–**19.01**	**0.018**
Mycotic infection	**17.67**	**1.65**–**189.16**	**0.018**
White blood cells	**1.05**	**1.00**–**1.10**	**0.041**
Bilirubin	**1.84**	**1.11**–**3.06**	**0.018**
AST_GOT	**1.01**	**1**–**1.008**	**0.018**
ALT_GPT	**1.005**	**1–1.01**	**0.03**
GGT	**1.006**	**1.002–1.009**	**0.002**
INR	2.46	0.28–21.68	0.418
CRP	**1.06**	**1.003–1.13**	**0.04**
Creatinine	2.02	0.85–4.83	0.112
Adherence	1.08	0.31–4.41	0.908
Conservative	0.96	0.27–3.42	0.953
Interventional	1.40	0.40–4.96	0.602
Surgery	-		-
Escalation	0.4	0.11–1.49	0.172

## Discussion

PLA is a complex disease as it is challenging to diagnose and treat correctly, still going along with high mortality of 18.2%. The results from our cohort are congruent to the described mortality in literature, which ranges from 10 to 40% [18].

Diagnosis of PLA can be difficult for the treating physician as symptoms are mainly unspecific. The leading symptom present in 90% of patients is fever and possibly chills. Local symptoms like abdominal pain are only present in circa 50% of cases [19]. Accordingly, laboratory findings often suggest inflammation. 97% of patients show an elevated CRP, but only about 50% have a pathological WBC. An elevated GGT is expected in about 80% of patients and can be a strong hint for a hepatic infectious focus, leading to hepatic imaging. Other pathological lab values are relatively infrequent. Only about 40% of patients present elevated liver aminotransferases. CRP and GGT are prognostic for PLA mortality. Therefore, we encourage focusing on both lab values to assess PLA risk. A good question for further research is how far these values can also monitor therapy success.

Diagnosis can then be made via medical imaging techniques, while both sonography and computer tomography (CT) are viable options. Interventional puncture or drainage of the PLA serves both diagnostic and therapeutic purposes, as the aspirated pus should undergo a microbiological examination to find the most fitting antibiotic agent [19]. Further surrounding diagnostics can be helpful to determine the pathogenetic cause of PLA. Examples are biliary MRI for the detection of bile duct obstruction or colonoscopy to diagnose intestinal pathologies [20].

A specific accumulation of cases with cirrhosis or diabetes cannot be seen in our cohort. This study is a representative sample of actual patients with PLA and is not designed to identify specific risk factors. However, other studies in the literature assessed diabetes [12] and cirrhosis [11] as risk factors for PLA.

In our study, only cases with primary unclear liver abscess were included. In 55%, a direct cause could be identified later, and only 45% remained cryptogenic. Therefore, we encourage further diagnostics like colonoscopy and MRI of the liver in cases of unclear PLA. Mainly the role of colonoscopy has been discussed and examined in the literature recommending inclusion in standard diagnostic for primarily unknown PLA [21].

The differential diagnosis for PLA must include particular causative pathogens: Amoebiasis is common in certain endemic regions and causes a liver abscess [22]. Also, hydatid cysts in echinococcus infection might superinfect and present as liver abscesses [23, 24]. Specific serological tests for these pathogens should always be performed in primarily unknown PLA.

In our cohort, treatment with antibiotics alone showed a failure rate of 82% and still 67% in patients with singular abscess smaller than 3 cm. Additionally, patients with conservative treatment had a significantly more extended hospital stay. Radiologically guided PLA puncture is the standard therapeutic option. Insertion of a drainage catheter is widely discussed with good evidence for both drainage insertion and singular needle aspiration [25]. Most patients in our study received primary drainage therapy (with catheter insertion), which was successful in about 70%. The remaining patients needed surgery later. While the literature recommends conservative treatment for small, singular PLA [17, 18], antibiotics alone appear insufficient as PLA treatment considering the present results. Past studies suggest that in more than 90% of cases, sufficient therapy can be reached by antibiotics in combination with drainage [18].

Multiple lesions present a unique therapeutic challenge, and primary surgery is recommended by some authors [17]. Non-surgical treatment was successful in about 49% of patients with multiple PLA in our study and 39% receiving surgery in the later course of their disease. Mortality was high, with 22% in both surgical and non-surgical treatment. As no patients with multiple lesions received primary surgery in our cohort, we cannot decide whether patients profit from immediate surgery. Based on the present data, we believe that multiple lesions can be treated without surgery if sufficient interventional drainage is technically possible.

**Fig. 2:**
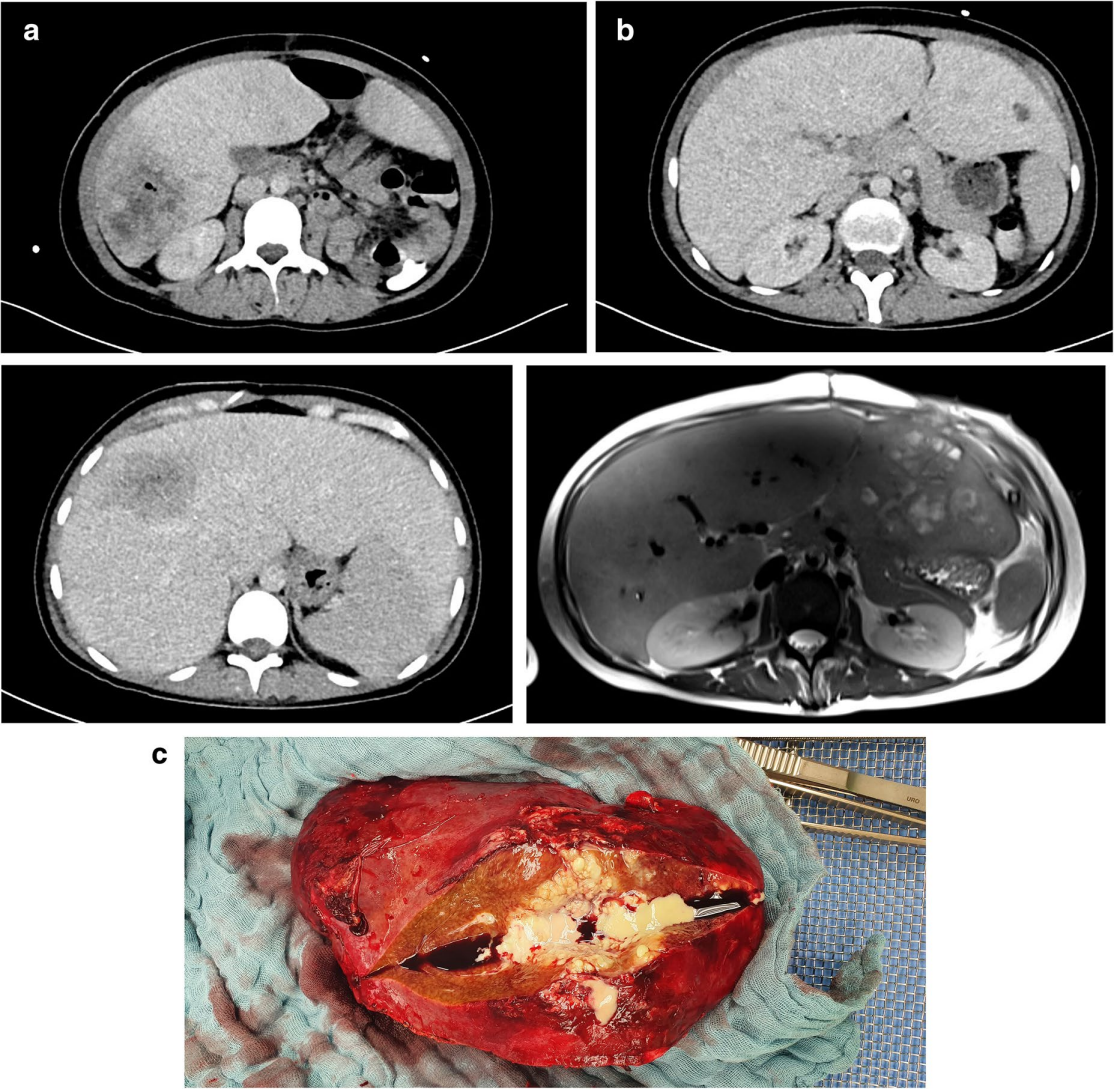
Panel **a** shows the initial CT of a female 29-year-old patient with multiple located PLA and inflammatory bowel disease. An interenteric fistula could be found as the infectious origin. Colon segment resection, repeated interventional drainage, and long-term antibiotic treatment were performed over 7 weeks. Lastly, PLA was narrowed to the left-lateral liver lobe (Panel **b**), and liver resection was performed in a final step (Panel **c**). The patient regenerated fully and had no PLA recurrence. This underlines the complexity of PLA treatment and the usefulness of interdisciplinary cooperation

Surgery showed a mortality of 12% and thus lower mortality than the overall cohort. Surgical drainage can be performed if interventional drainage fails or is anatomically impossible (e.g., intestinal interposition making drainage technically challenging), while the latter should be preferred [26]. Resection in patients with PLA goes along with slightly higher mortality than hepatectomy in oncological patients [27], even when performed in hepatobiliary centers. Similar results are described by other experienced hepatobiliary surgeons [16, 28]. This underlines the importance of interdisciplinary collaboration between treating physicians, interventional radiologists, and hepatobiliary surgeons (Fig. 2).

Concluding our data, we cannot encourage the proposed treatment protocol by Hope et al. and others citing it [17, 18]. Adherence to the treatment protocol did not influence PLA mortality. Conservative treatment (with antibiotics alone) appears obsolete, with a high failure rate of about 82%. Drainage should always be aimed for. Pang et al. report a resembling experience at their center [29]. Interventional drainage in terms of radiologically guided puncture is superior to surgical drainage [26]. Even in multiple localized PLA, drainage can be promising. In our cohort, patients always had a drainage catheter inserted, while the literature suggests that a single aspiration can be just as effective [25]. Surgery plays a vital role in failed or anatomically impossible drainage cases and should only be performed by the experienced hepatobiliary surgeon considering the persisting high mortality [16].

**Table:** 

Management of primarily unknown liver abscess 1. Always aspire drainage a. Preferably interventional drainage b. In case of technically not possible drainage evaluate surgery, if necessary contact hepatobiliary center c. Only refrain from drainage, if technically not possible and patient is unfit for surgery
2. Empiric antibiotic therapy, later adjust to microbiological findings 3. Further diagnostics: colonoscopy, MRI/MRCP, serological tests 4. If not responding in terms of symptoms and lab values: evaluate surgery

## Conclusion

-
  - Primarily unknown liver abscesses go along with a high mortality of 18%. Abscesses with multiple localizations are particularly challenging.
-
  - Symptoms are unspecific. Diagnosis can be made using imaging techniques. CRP and GGT are both sensitive for PLA and are correlated with the individual risk.
-
  - Surrounding diagnostics like colonoscopy and MRI are helpful as in 55% of patients; a causative condition like colon cancer, previous diverticulitis, or bile duct pathologies can be found.
  - Radiologically guided drainage and antibiotics are the therapy of choice and are successful in 70% of cases.
  - If drainage is insufficient or impossible, surgery should be performed. The affected segment’s surgical drainage and resection show promising results if performed by an experienced hepatobiliary surgeon.

## Lay summary

Liver abscess is a collection of pus in the liver with an unclear cause. A reason for the abscess can only be found in half of patients. Liver abscesses are dangerous conditions with a mortality of 18%. The therapy of choice is the evacuation of pus via drainage or surgery.

## Abbreviations

PLA: Pyogenic liver abscess
CRP: C-reactive protein
GGT: Gamma-glutamyl-aminotransferase
pPLA: Primary pyogenic liver abscess
INR: International normalized ratio
